# 2. Case scenarios 1: Trauma based approach: from instability to stability

**DOI:** 10.1192/j.eurpsy.2023.61

**Published:** 2023-07-19

**Authors:** F. Baessler

**Affiliations:** Psychosomatics, University Clinic and Heidelberg Academy of Sciences and Humanities, Heidelberg, Germany

## Abstract

**Abstract:**

This presentation is titled " Case scenarios 1: Trauma based approach: from instability to stability". You will be given a case based oriented insight into the relationship between substance use disorder and forced displacement and treatment options that are based upon the trauma some has been experienced. A focus will be on factors that contribute on psychological stability in extreme situations. Thus you will be presented preliminary results of our ongoing study funded by the Heidelberg Academy of Sciences and Humanities that dedicated to the question of how mental stability and health-related quality of life of individuals change over time.

**Disclosure of Interest:**

None Declared

